# Overall and Anterior Tooth Size Ratios in a Group of Emiratis

**DOI:** 10.2174/1745017901814010655

**Published:** 2018-09-28

**Authors:** Moaza Ghuloom Mohammad, Shazia Naser-ud Din, Amar Hassan Khamis, Athanasios E. Athanasiou

**Affiliations:** 1 Department of Orthodontics, Hamdan Bin Mohammed College of Dental Medicine, Mohammed Bin Rashid University of Medicine and Health Sciences, Dubai, United Arab Emirates; 2 Orthodontic Clinic, Al Mizhar Health Center, Primary Health Care Center, Dubai Health Authority, Dubai, United Arab Emirates; 3 Department of Biostatistics, Hamdan Bin Mohammed College of Dental Medicine, Mohammed Bin Rashid University of Medicine and Health Sciences, Dubai, United Arab Emirates

**Keywords:** Bolton tooth size analysis, Overall and anterior tooth size ratios, Emiratis, Class I normal occlusion, Class II malocclusion, Orthodontic treatment

## Abstract

**Objectives:**

The aims of this investigation in a group of Emiratis were (**1**) To study overall and anterior tooth size ratios in Class I normal occlusion, (**2**) To estimate overall and anterior tooth size ratios in different malocclusion groups, (**3**) To compare overall and anterior tooth size ratios in Class I normal occlusion with the Bolton standards, and (**4**) To determine the distribution of overall and anterior tooth size ratios ± 2 SD from Bolton mean values in all occlusion groups.

**Materials and Methods:**

In this cross-sectional investigation, consecutive patients’ files, including dental casts, were selected from the archives of orthodontic clinics of the Dubai Health Authority. The final sample was formed following the application of specific inclusion criteria. The sample consisted of 521 pairs of dental casts representing both sexes (males: 188; females: 333) and different malocclusion groups (Class I: 288; Class II: 110; Class III: 30) and Class I normal occlusion (93). The mean age of patients was 16.18y for Class I, 15.73y for Class II, 15.83y for Class III, and 16.55y for Class I normal occlusion. The dental casts were scanned and digitized by the first author using the Ortho Insight 3D laser scanner. Measurements were made regarding maxillary and mandibular sums of mesiodistal tooth dimension of the overall (6-6) and anterior (3-3) groups of teeth. Statistical analysis included descriptive statistics, paired *t*-test and Analysis of Variance (ANOVA). The level of significance was set at *p*<0.05.

**Results:**

There were statistically significant differences among malocclusion groups with regard to overall and anterior tooth size ratios. However, the comparison between the Class I normal occlusion group and the Bolton standards showed no statistically significant differences. Only five cases in Class II malocclusion presented an anterior tooth size discrepancy outside plus 2 SD from Bolton mean values and one case in Class I malocclusion presented with an overall tooth size discrepancy outside plus 2 SD from Bolton mean values.

**Conclusion:**

This study of the different occlusion groups of the Emirati sample concluded that (**a**) Class I normal occlusion cases presented similar overall and anterior tooth size ratios to Bolton standards; (**b**) Overall and anterior tooth size ratios among different malocclusion groups exhibited statistically significant differences; (**c**) Five cases in Class II malocclusion presented an anterior tooth size discrepancy outside plus 2 SD from Bolton mean values, and (**d**) One case in Class I malocclusion presented an overall tooth size discrepancy outside plus 2 SD from Bolton mean values.

## 1 INTRODUCTION

After completing orthodontic treatment, specific dimensional relationships should exist between the maxillary and mandibular teeth to ensure ideal conditions of interdigitation, overjet and overbite [ 1 ] . Since natural teeth match well in most individuals, tooth size discrepancy of less than 1.5 mm is rarely significant, but larger discrepancies create treatment problems and must be included in the orthodontic problem list [ 2 ] . Patients with interarch tooth size discrepancies typically require special finishing steps, such as enamel removal (interproximal reduction) or crown material addition (composite buildups or porcelain veneers), to correct this discrepancy so that the teeth occlude harmoniously. Good occlusion needs proportional tooth size relationships between dental arches.

Tooth size analysis, the degree of disproportional relationships between upper and lower teeth (in total or anterior) with regard to the mesiodistal dimension, often called Bolton analysis after its developer, is carried out by measuring the mesiodistal width of each permanent tooth excluding second and third molars [ 3 ] . Bolton concluded that the normal ratio for physiologic occlusion is an overall ratio of 91.3+1.91 and an anterior ratio of 77.2+1.65 [ 4, 5 ] . 

Laser scanned digital models are highly accurate compared to physical models and Bolton analysis can be accurately and reliably performed in this digital format [ 6 ] . 

Although much research has been carried out to document tooth size discrepancy in different malocclusions, gender, populations and ethnic/racial groups [ 7 ] , no relevant data is available for Emiratis.

The aims of the present investigation in a group of Emiratis were (**1**) To study overall and anterior tooth size ratios in Class I normal occlusion, (**2**) To estimate overall and anterior tooth size ratios in different malocclusion groups, (**3**) To compare overall and anterior tooth size ratios in Class I normal occlusion with the Bolton standards, and (**4**) To determine the distribution of overall and anterior tooth size ratios ± 2 SD from Bolton mean values in all occlusion groups.

## 2 MATERIALS AND METHODS

The study received ethical approval from the Hamdan Bin Mohammed College of Dental Medicine and the Dubai Health Authority.

This is a cross-sectional study utilizing complete orthodontic records including study plaster models. The files of consecutive patients, who had been treated from January 2005 until December 2015, including pre-treatment and post-treatment dental casts, were selected from the archives of the orthodontic clinics of the Dubai Health Authority, located in Bur Dubai (Al Badea Dental Center and Al Barsha Health Center) and Bur Deira (Nad Al Hamar Health Center, Dubai Hospital and Al Mizhar Health Center), Dubai, United Arab Emirates.

Sample selection was undertaken following application of certain inclusion criteria: Healthy patients, aged 13-18y; Emirati origin having a United Arab Emirates passport and Emirate identification document; complete permanent dentition, but with second and third molars allowed to be absent; normal tooth crown form; no dental anomalies in tooth number; no history of interproximal reduction; no restorations altering mesiodistal tooth crown width; complete record files; and excellent quality of study models.

Thus, 521 patients met the criteria (males: 188; females: 333), with 428 pretreatment and 93 posttreatment pairs of dental casts divided according to Angle classification into three malocclusion groups and a Class I normal occlusion group, respectively. The latter group was characterized by Class I molar and canine relationships as well as physiologic overjet, overbite, alignment and leveling.

Table **1** presents the characteristics of the different groups of patients according to occlusion, gender and age.

All maxillary and mandibular dental casts were scanned by the first author using an Ortho Insight 3D laser scanner (3D Motion View, Chattanooga, Tennessee, USA) with scanning resolution being set to “high” using the Ortho Insight 3D Software (Version 6.0.7044). Measurements were made regarding maxillary and mandibular sums of mesiodistal tooth dimension of the overall (6-6) and anterior (3-3) groups of teeth. Following scanning of all models, the digitization and assessment of the variables of their electronic version included the following stages: **(1)** “Separate teeth” using Ortho Insight 3D, so that each model was separated and each tooth was marked individually; **(2)** “Detect landmarks” without performing changes in the major anatomically detected landmarks in the dentition, so that 21 landmarks were automatically produced on each tooth by the Ortho Insight 3D Software (buccal cusp, gingival buccal groove, occlusal buccal groove, cementoenamel junction, central fossa, central incisal, central labial, cusp, distal pit, distobuccal cusp, distoincisal, distolabial, distolingual cusp, distal marginal ridge, lingual cusp, mesial pit, mesiobuccal cusp, mesioincisal, mesiolabial, mesiolingual cusp, mesial marginal ridge), and **(3)** “Set FAs (Facial Axes) and measure teeth” so that the mesial and distal contact points of each tooth were adjusted and checked using the tooth view window, which had occlusal, facial and mesial views provided by the software.

Final data included patient’s information (name, ID, model number, birth date and age when the records were taken), sum of mesiodistal dimension of upper and lower twelve teeth (6-6) and sum of mesiodistal dimension of upper and lower six anterior teeth (3-3), and overall and anterior ratios. All data were entered into an Excel file and patients’ confidentiality was ensured.

The previously mentioned sample size was calculated using Cochrane analysis formula that provides sample size and power analysis for the hypothesis test, confidence interval and equivalence analysis [ 8, 9 ] . A total sample size of 595 subjects was calculated and power of 80%, with 95% significance level was given utilizing the following formulas:

$$N=\frac{Z_{\frac{\alpha }{2}}^{2}\mathrm{pq}}{B^{2}}$$

Where Zα/2 is the quartile of 95% of the confidence interval

P is the prevalence of the disease under study population and q is (1- p)

B is precision or (margin error) and is given by

$$B=\sqrt{\frac{Z_{\alpha /2}}{n}}\mathrm{pq}$$

Where “n” is the sample size from which the prevalence p was calculated.

Data were elaborated using the special statistical program *SPSS* (Statistical Package for Social Science for Windows Version 20.0, *SPSS* Inc., Chicago, Illinois, USA). Measurements per variables were tested for normality by using the Kolmogorov-Smirnov test, which was applied among different occlusion groups. The cross-tabulation test was used to examine the independency between categorical variables. Where two or more continuous independent variables were examined, the Student’s *t*-test and Analysis of Variance (ANOVA) were used, respectively. A *p*-value of less than 0.05 was considered significant in all statistical analyses.

In order to test intra-examiner reliability, the first author re-measured 50 pairs of models, randomly selected from the original sample, one week after the initial measurements. The intra-examiner reliability was assessed using the paired *t*-test, and no statistically significant difference between the first and second measurements was found, thus confirming intra-examiner reliability ( Table **2** ). 

## 3 RESULTS

The results of testing for normality using the Kolmogorov-Smirnov test revealed that most of the variables were normally distributed and because the sample size within each occlusion group was large (≥30) parametric statistics were chosen for analyzing the data.

Table **3** presents the descriptive statistical data of all variables among the four occlusion groups as well as the results of the comparisons between mean values of overall and anterior tooth size dimensions and ratios among different occlusion groups. Statistically significant differences (*p*<0.001) were found in all variables of the various occlusion groups except the maxillary 6-6 sum (*p*=0.148).

No statistically significant differences in overall and anterior tooth size ratios were found in relation to gender (Table **4**) and between the normal occlusion Emirati group and the Bolton standards (Table **5**). 

There were statistically significant differences (*p*<0.001) in overall and anterior tooth size ratios among the different malocclusion groups (Table **6**). 

The frequency of tooth size discrepancy outside 2 SD from the Bolton mean values for overall and anterior ratios were calculated for all occlusion groups. Table **7** shows the distribution of cases with anterior tooth size discrepancies outside 2 SD from the Bolton mean values (anterior ratio $\overset{}{X\;}$= 77.2 ± 2 SD: 73.9 and 80.5, respectively). Five cases in Class II malocclusion presented anterior tooth size discrepancy outside plus 2 SD from the Bolton mean values. Table **8** shows the distribution of cases with overall tooth size discrepancies outside 2 SD from the Bolton mean values (overall ratio $\overset{}{X\;}$= 91.3 ± 2 SD: 87.48 and 95.12, respectively). There was one case of Class I malocclusion with an overall tooth size discrepancy outside plus 2 SD from the Bolton mean values.

## 4 DISCUSSION

Although tooth size discrepancy with regard to overall and anterior ratios has been studied in a significant number of ethnic and racial groups as well as malocclusion types and gender, such an investigation has not been undertaken for the Emirati population. Therefore, it is valuable to investigate the tooth size overall and anterior ratios in a group of Emiratis and to compare their values with the Bolton standards.

When plans for forming the Class I normal occlusion group were being made, it was realized that the sample size required by the power analysis calculation (N = 180) would be difficult to be formed. Individuals with ideal or normal occlusion in such numbers cannot be easily identified, recorded and studied in any population. Therefore, as an alternative, the group of Class I normal occlusion was formed by utilizing orthodontic post-treatment patients’ study casts. Special selection criteria were applied in selecting these cases so that factors of tooth morphology and orthodontic treatment characteristics did not influence the mesiodistal dimensions of the teeth which were measured. This is the reason that in the present study 521 consecutive cases were included, this being less than the ideal number of 595 of the sample size calculation.

The inclusion criterion of Emirati background was checked and assessed utilizing administrative data from patients’ files of the Dubai Health Authority.

The traditional way for study model analysis involves plaster casts measured with calipers. However, in recent years, three-dimensional virtual study models have become increasingly used in dentistry. The diagnostic accuracy and measurement sensitivity of electronic models compared to plaster models have been thoroughly investigated. In a study focusing on comparisons between measurements on digital models and measurements with digital calipers on plaster models, it was concluded that “digital models offer a high degree of validity when compared to direct measurement on plaster models” [ 10 ] . Based on this conclusion, the decision was made to perform the present investigation using the Ortho Insight 3D scanner with the resolution set in “high” and utilizing the dedicated software provided. The digital models subsequently produced and elaborated were subject to Auto Bolton analysis with the same software.

Many studies have compared orthodontic tooth measurements using digital casts and plaster models. Tooth width measurements on digital models can be as accurate as, more reproducible, and significantly faster than those taken on plaster models [6, 11 ] . When the diagnostic accuracy and surface matching characteristics of three-dimensional digital dental models obtained from various sources were studied, it was concluded that all of them can provide diagnostic information similar to caliper measurements, with varying degrees of agreement limits. The scanner virtual model has the least mean bias. A strong surface match correlation was observed between virtual scanned and electronic models, thus indicating that these could be used interchangeably. It is important to note that from the numerous scanning and software systems previously tested, the Ortho Insight 3D scanner and software used in this study have been proved to be very accurate [6, 12 ] .

Several other investigations have compared the findings from plaster casts and those from electronic model measurements reporting dental arch dimensions, Bolton index, space analysis and irregularity index. A good reliability of correspondence between both records was found in almost all parameters regarding three dimensions for both digital and plaster models compared with plaster models [13-15].

To confirm the accuracy and consistency of the operator’s measurements, the second series of measurements for all variables was conducted with the author/operator blinded for a group of randomly selected sets of models. The results showed consistency between the two series of measurements.

In the present study, where the sample of post-treatment normal occlusion group was compared to the Bolton standards, the results showed that the mean values and standard deviations of anterior and overall ratios obtained were not statistically significantly different. The absence of statistically significant differences in both ratios of this group with the Bolton values was further reinforced with the very small number of cases presenting values outside the two SD ranges. Only five cases in Class II malocclusion presented an anterior tooth size discrepancy outside plus 2 SD and one case in Class I malocclusion presented with an overall tooth size discrepancy outside plus 2 SD, from Bolton mean values respectively.

Although the normal Class I occlusion group in the present study consisted of orthodontically treated cases with optimal occlusal features and full sets of teeth, it has to be conceded that this is not identical to a normal untreated sample. Obviously, the great difficulties in obtaining records of a normal occlusion sample led to the abandoning of this option and resorting to the use of orthodontically treated cases. On the other hand, it should be recognized that the strict inclusion criteria implemented for the formation of the present normal occlusion group excluded cases with altered crown morphology resulting from previously performed orthodontic treatment. In addition, all cases with pathological, developmental or therapeutic aspects influencing crown morphology were not included in this sample.

Based on existing findings in the relevant literature, absence of significant differences with the Bolton standards has been found in other populations from the Middle East region [16-23]. The fact that most of the populations surveyed in these studies have Caucasian backgrounds may explain the similarity with the North American Caucasian sample used in the Bolton report.

When anterior and overall tooth size ratios were compared among the four occlusion groups and the three malocclusion groups, respectively, statistically significant differences were found between the groups as has been reported elsewhere [24-31]. Increased values of an overall Bolton ratio for Class III malocclusion patients have been previously reported [25, 28]. This finding is not unexpected given that one of the etiological factors of malocclusion is deviation in tooth size - jaw size relationships.

No sexual dimorphism was found when overall and anterior tooth size ratios were studied regarding gender.

Based on the finding of this study, implementation of a detailed, comprehensive and goal-oriented treatment plan in Emirati patients requiring orthodontic therapy should take into account that our group did not exhibit different anterior and overall tooth size ratios from Bolton values. However, there is a possibility that individual variations may exist and this should be considered.

## CONCLUSION

This study of the different occlusion groups of the Emirati sample concluded that **(a)** Class I normal occlusion cases presented similar overall and anterior tooth size ratios to Bolton standards; **(b)** Overall and anterior tooth size ratios among different malocclusion groups exhibited statistically significant differences; **(c)** Five cases in Class II malocclusion presented an anterior tooth size discrepancy outside plus 2 SD from Bolton mean values, and **(d)** One case in Class I malocclusion presented an overall tooth size discrepancy outside plus 2 SD from Bolton mean values.

## Figures and Tables

**Table 1:** Characteristics of the different groups of patients according to occlusion, gender and age.

-	Class I	Class II	Class III	Normal	Total
Female	181 (54.4%)	67 (20.1%)	20 (6%)	65 (19.5%)	333 (100%)
Male	107 (56.9%)	43 (22.9%)	10 (5.3%)	28 (14.9%)	188 (100%)
Mean age (SD)	16.18 (1.8) y	15.73 (1.8) y	15.83 (1.9) y	16.55 (1.6) y	-

**Table 2:** Reliability test of consistency for readings of investigator by using paired *t*-test (R1 indicates first measurement and R2 indicates second measurement).

Measurements	Mean Differences (mm)	Std. Error (mm)	95% CI		*p* value
			Lower	Upper	
maxillary 3-3 R1 - maxillary 3-3 R2	-0.1316	0.10144	-0.33545	0.07225	0.201
mandibular 3-3 R1 - mandibular 3-3 R2	-0.1066	0.081093	-0.26956	0.056362	0.195
anterior ratio R1 - anterior ratio R2	-0.0102	0.006024	-0.02231	0.001905	0.097
maxillary 6-6 R1 – maxillary 6-6 R2	-0.1128	0.09691	-0.30755	0.08195	0.25
mandibular 6-6 R1 - mandibular 6-6 R2	-0.0294	0.09318	-0.21665	0.15785	0.754
overall ratio R1 - overall ratio R2	0.0738	0.05087	-0.02843	0.17603	0.153

**Table 3:** Descriptive statistics of overall and anterior tooth size dimensions and ratios among different occlusion groups and comparisons of their mean values by ANOVA.

Classification		Maxillary 3-3 (mm)	Mandibular 3-3 (mm)		Anterior ratio (%)	Maxillary 6-6 (mm)	Mandibular 6-6 (mm)	Overall Ratio (%)
Class I	N	288	288		288	288	288	288
	Mean	46.92	36.62		78.05	98.45	90.18	91.57
	SD	0.70	0.53		0.52	1.29	1.28	0.38
	Min	45.3	35.27		77.13	94.28	86.16	91.13
	Max	49.69	38.72		79.42	103.51	98.62	97.01
	SE	0.04	0.03		0.03	0.07	0.07	0.02
Class II	N	110	110		110	110	110	110
	Mean	48.88	38.64		79.14	100.32	91.83	91.54
	SD	1.74	1.38		0.72	3.03	2.82	0.50
	Min	44.28	34.19		77.78	91.29	83.64	90.06
	Max	54.2	42.56		82.13	107.90	99.06	92.74
	SE	0.17	0.13		0.07	0.29	0.27	0.05
Class III	N	30	30		30	30	30	30
	Mean	48.25	37.40		77.54	98.7	89.03	90.21
	SD	1.75	1.32		0.66	3.42	3.26	0.79
	Min	44.5	34.3		76.18	90.86	80.98	89
	Max	52.29	40.14		78.84	104.2	94.39	91.79
	SE	0.32	0.24		0.13	0.62	0.60	0.14
Normal	N	93	93		93	93	93	93
	Mean	48.36	37.46		77.54	99.37	90.85	91.41
	SD	2.0	1.42		0.30	2.87	2.61	0.22
	Min	44.24	34.29		77.03	92.22	84.38	91
	Max	55.10	41.28		79.24	106.77	97.91	91.99
	SE	0.21	0.15		0.03	0.30	0.30	0.02
*p* value	-	0.001	0.001		0.001	0.148	0.001	0.001

**Table 4:** Comparison of overall and anterior tooth size ratios according to gender (*t*-test).

Ratio	Gender	N	Mean (SD)	*p* value
Anterior ratio (%)	Female	333	78.17 (0.77)	0.655
	Male	188	78.14 (0.77)	
Overall ratio (%)	Female	333	91.46 (0.56)	0.633
	Male	188	91.44 (0.45)	

**Table 5:** Comparison of overall and anterior tooth size ratios between the normal occlusion Emirati group and the Bolton standards (t-test).

Ratios	Normal				
	Present Study		Bolton Standard		*p* value
	N	Mean (SD)	N	Mean (SD)	
Anterior ratio	93	77.54 (0.30)	55	77.2 (1.65)	0.340
Overall ratio	93	91.41 (0.22)	55	91.3 (1.91)	0.110

**Table 6:** Comparison of overall and anterior tooth size ratios among different malocclusion groups (ANOVA).

Classification	N	Mean (%)	SD (%)	SE	*p* value
Anterior ratio					
Class I	288	78.05	0.52	0.03	0.001
Class II	110	79.14	0.72	0.07	
Class III	30	77.54	0.66	0.13	
Overall ratio					
Class I	288	91.56	0.38	0.02	0.001
Class II	110	91.54	0.50	0.05	
Class III	30	90.2	0.79	0.14	

**Table 7:** Distribution of cases with anterior tooth size discrepancies outside 2 SD from Bolton mean values (anterior ratio $\overset{-}{X\;}$= 77.2 ± 2 SD: 73.9 and 80.5, respectively).

-	Anterior Ratio						
Classification	Outside SD (%)	SD 2 (%)	SD 1 (%)	Mean (%)	SD 1 (%)	SD 2 (%)	Outside SD (%)
-	< 73.9	73.9-75.4	75.5-77.1	77.2	77.3-78.8	78.9-80.5	>80.5
Class I	-	-	-	-	250 (94)	16 (6)	-
Class II	-	-	-	-	37 (35.6)	62 (59.6)	5 (4.8)
Class III	-	-	8 (29.6)	-	19 (70.4)	-	-
Normal	-	-	3 (3.7)	1 (1.2)	76 (94)	1 (1.2)	-

**Table 8:** Distribution of cases with overall tooth size discrepancies outside 2 SD from Bolton mean values (overall ratio $\overset{-}{X\;}$= 91.3 ± 2 SD: 87.48 and 95.12, respectively).

-	Overall Ratio						
Classification	Outside SD (%)	SD2 (%)	SD 1 (%)	Mean (%)	SD 1 (%)	SD 2 (%)	Outside SD (%)
-	< 87.4	87.4-89.3	89.4-91.2	91.3	91.4-93.2	93.3-95.1	>95.1
Class I	-	-	1 (0.4)	1 (0.4)	222 (98.7)	-	1 (0.4)
Class II	-	-	15 (17.6)	1 (1.2)	69 (81.2)	-	-
Class III	-	3 (11.5)	21 (80.8)	-	2 (7.7)	-	-
Normal	-	-	16 (25.8)	-	46 (74.2)	-	-
